# A Quasi-experimental Study on the Use of Retrospective Case Construction to Teach Hepatobiliary Function Test Interpretation to First-Year Medical Students

**DOI:** 10.7759/cureus.104082

**Published:** 2026-02-22

**Authors:** Palani Selvam Mohanraj, Jancy K Jose, Niranjan Gopal, Amle Dnyanesh Balkrishna, Roshan Takhelmayum, Utsav Haldar, Jyoti E John, Anand Dixit

**Affiliations:** 1 Department of Biochemistry, All India Institute of Medical Sciences, Gorakhpur, Gorakhpur, IND; 2 Department of Biochemistry, All India Institute of Medical Sciences, Nagpur, Nagpur, IND; 3 Department of Community Medicine and Family Medicine, All India Institute of Medical Sciences, Gorakhpur, Gorakhpur, IND

**Keywords:** active learning strategies, case-based teaching, clinical biochemistry, early clinical exposure, knowledge retention, retrospective case construction

## Abstract

Background

The inclusion of early clinical exposure (ECE) in the medical curriculum has emphasized the importance of developing laboratory interpretation skills among MBBS students. Clinical biochemistry, a critical component of evidence-based medicine, promotes students’ understanding of clinical issues. This study examined the impact of biochemical report interpretation as a pedagogical technique for first-year medical students, focusing on hepatobiliary function tests. It analyzes the short- and long-term retention of knowledge and assesses students' perceptions of this method.

Methods

A single-group, pre-test/post-test quasi-experimental study with a delayed post-test was conducted. Group-based learning was conducted with 120 students following a didactic presentation on hepatobiliary function tests. Assessments were conducted via a pre-test, post-test, and delayed post-test four weeks later using a structured questionnaire with multiple-choice and short-answer questions. Students created retrospective case narratives based on five laboratory results. Data analysis included statistical comparisons of the scores and input from students and facilitators.

Results

Post-test scores showed significant improvement in students' interpretation skills, particularly in recognizing obstructive jaundice. The delayed post-test scores revealed satisfactory long-term retention. Feedback highlighted the impact of this strategy in promoting conceptual understanding and improving diagnostic accuracy.

Conclusions

Retrospective case construction significantly improved hepatobiliary function test interpretation skills in first-year medical students, with immediate post-test gains sustained at four weeks.

## Introduction

In contemporary medical education, introducing practical, clinically oriented learning experiences early in the curriculum is crucial for equipping students with the necessary competencies for clinical decision making and patient care [1]. Early clinical exposure (ECE) has been shown to enhance students' understanding of basic science and clinical applications. For example, 43% of students strongly agreed that ECE influenced their perspective on learning pre-clinical topics, while 46.6% strongly agreed that it helped them better understand the issue [2]. Similarly, another study indicated that ECE improved students' understanding of the practical applications of physiology [3]. By teaching clinical principles and practical applications in the early years of medical school, ECE encourages critical thinking, contextual knowledge, and the ability to connect core science with clinical scenarios [4]. This pedagogical approach has gained considerable popularity in recent years, especially in disciplines like clinical biochemistry, where laboratory data interpretation plays a crucial role in the identification and control of diseases. 

Clinical biochemistry is the cornerstone of clinical medicine, providing insights into the physiological and pathological conditions of the human body through biochemical investigations. Among the many laboratory tests, hepatobiliary function tests are of particular value because of their ability to measure the health of the liver and biliary tract, which is a critical area in clinical practice [5]. These tests provide important diagnostic markers for diseases such as obstructive jaundice, liver dysfunction, and hemolytic disorders [6]. However, despite their relevance, understanding these complex reports often presents challenges for medical students who may lack the analytical skills and clinical context to derive valuable insights from the data. 

The gap between theoretical teaching and practical applications often leads to knowledge gaps, limiting students' ability to effectively integrate biochemistry into clinical situations. To address these challenges, case-based learning (CBL) and laboratory report interpretation exercises have been developed as effective pedagogical tools in medical education. CBL emphasizes the application of academic knowledge to realistic scenarios, helping students acquire problem-solving skills in simulated clinical environments [7]. Retrospective case construction, a specific variant of CBL, further expands this approach by requiring students to study laboratory data and "work backwards" to develop hypothetical patient histories, including symptoms, signs, and a provisional diagnosis. This version aligns well with the goals of CBL, which include developing critical thinking, improving higher-level learning outcomes, and enhancing problem-solving abilities [8]. By developing case histories by working backward from laboratory data, students are challenged to apply their knowledge in a more complex and realistic manner. This technique can be particularly effective in developing students' clinical reasoning skills, which are crucial for physicians [9]. 

This study focused on the use of retrospective case construction from hepatobiliary function test reports as a teaching method for first-year medical undergraduate students. First-year students are the focus because they are in the phase of learning foundational biochemical concepts. Introducing this method at this stage helps bridge the gap between theory and clinical application early in their training (ECE), building a strong foundation for their future clinical years. By introducing this strategy early in the medical curriculum, this study aimed to enhance students' ability to understand laboratory data, link biochemical results to clinical diagnoses, and retain basic concepts over time. The selection of hepatobiliary function tests as the focus of this study reflects their diagnostic value and the common obstacles students face when interpreting these data. The primary outcome measured was the change in students' interpretation skills and knowledge retention, as assessed by test scores. Furthermore, the integration of structured feedback and iterative assessments, including pre-tests, post-tests, and delayed post-tests, provided a comprehensive framework for evaluating the outcomes of this teaching style. 

In this study, we address this essential gap in medical education by focusing on the adoption of ECE through retrospective case-building as a strategy to enhance students' analytical and interpretative abilities necessary for clinical practice. By emphasizing the interpretation of hepatobiliary function tests, this study provides a formal framework for evaluating the success of this teaching strategy. The findings highlight that this method could serve as a model for broader curricular reforms in biochemistry and allied sciences, encouraging a more integrated and clinically relevant learning experience for medical students.

## Materials and methods

This study adopted a structured multi-phase strategy to analyze the effectiveness of retrospective case construction using hepatobiliary function test reports as a teaching and learning method for first-year medical students. The methodology was designed to measure short- and long-term information retention, enhance laboratory data interpretation skills, and gauge students' perceptions of this unique teaching strategy. The study components included didactic lectures, small-group learning sessions, and sequential assessments (Table 1).

**Table 1:** Study Design and Assessment Timeline MCQs: multiple-choice questions, ALP: alkaline phosphatase, ALT: alanine aminotransferase, AST: aspartate aminotransferase

Phase	Activity	Description	Timing
1	Pre-test	Assessment of baseline knowledge via MCQs and short-answer questions on hepatobiliary function tests.	Day 1 (Before intervention)
2	Didactic Lecture	Structured lecture covering biochemical markers (bilirubin, ALT, AST, ALP) and their interpretation in conditions like obstructive, hepatic, and hemolytic jaundice.	Day 1
3	Group-Based Learning	Students (n=120) divided into 16 small groups. Each group analyzed five hepatobiliary function test reports to construct retrospective case narratives. Facilitators provided guidance.	Day 1 (Immediately after lecture)
4	Post-test	Assessment identical to the pre-test to measure short-term knowledge gain.	Day 1 (After group activity)
5	Delayed Post-test	Assessment to measure long-term knowledge retention.	Four weeks after intervention

Study setting and ethical considerations

This quasi-experimental study was conducted over a six-week period in December 2025 at the All India Institute of Medical Sciences, Gorakhpur. First-year medical students from the Department of Biochemistry were recruited as participants. Prior to the commencement of the study, approval was obtained from the Institutional Human Ethics Committee (IHEC) (Approval Number: IHEC/AIIMS-GKP/BMR/632/2025), ensuring the protocol adhered to established ethical guidelines for educational research. Furthermore, informed consent was secured from all participating students, detailing the study's purpose, procedures, and their right to confidentiality and voluntary participation without any impact on their academic standing.

Participant selection

All 126 first-year medical students enrolled in the 2025 academic batch were eligible to participate in the study. Students who were absent on the day of the core educational intervention or any of the three assessment days (pre-test, post-test, delayed post-test) were excluded from the final data analysis. Students who did not provide written informed consent were also excluded. Six students were excluded from the final analysis due to absence on one or more assessment days. Therefore, a total of 120 students completed all three assessments and were included in the final data analysis.

Study population

A total of 120 first-year medical students enrolled in the 2025 academic batch at All India Institute of Medical Sciences, Gorakhpur, completed all three assessments and were included in the final analysis. The study population comprised 68 male (56.7%) and 52 female (43.3%) students, with an age range of 18 to 22 years (mean age 19.4 ± 1.2 years). All participants had completed their higher secondary education with biology as a core subject and were in the first semester of their MBBS curriculum at the time of the study. None of the participants had received prior formal training in hepatobiliary function test interpretation. The demographic characteristics of the study population are summarized in Table 2.

**Table 2:** Demographic Characteristics of Study Participants (N=120) Data are presented as number (n) and percentage (%). LFT: liver function test. Age was self-reported at the time of the study.

Characteristic	Category	n	%
Gender	Male	68	56.7
	Female	52	43.3
Age (years)	18-19	73	60.8
	20-22	47	39.2
	Mean ± SD	19.4 ± 1.2	—
Educational Background	Science (Biology)	120	100
Prior Training in LFT Interpretation	No	120	100

Study design

This study employed a single-group, pre-test/post-test quasi-experimental design with a delayed post-test. This design was chosen as it is a standard and ethical approach in educational research to measure the impact of a new curriculum intervention within a single cohort of students. It allows for the comparison of students' interpretation skills and knowledge at three critical time points: at baseline (pre-test), immediately after the educational intervention (post-test), and after a four-week interval to assess knowledge retention (delayed post-test). All 120 participants were exposed to the same intervention, which consisted of a didactic lecture followed by a group-based retrospective case construction activity focused on hepatobiliary function tests. The primary outcome was the change in mean assessment scores across the three time points.

Didactic lectures

The initial phase of this study included structured didactic lectures on hepatobiliary function tests. These lectures provided a theoretical framework for the subsequent practical activities. The lectures, delivered by an experienced academic member, covered the following: biochemical markers used in hepatobiliary function tests, including bilirubin (direct and indirect), alanine aminotransferase (ALT), aspartate aminotransferase (AST), alkaline phosphatase (ALP), and urinary bile pigments; the physiological and pathological factors underlying hepatobiliary diseases, including obstructive jaundice, liver dysfunction, and hemolysis; and interpretation strategies for test results and their connection with clinical presentations. The lecture aimed to provide students with an overview of the topic and prepare them for practical application through group discussions and case-based exercises. 

Group-based learning activities

Following the didactic presentation, students were divided into 16 small groups, each supervised by a faculty member. The group size was maintained to encourage active participation and customized guidance. Facilitators who were previously trained in the study objectives and format ensured uniformity of delivery across all groups. Each group was presented with five hepatobiliary function test reports covering a range of clinical conditions, including obstructive jaundice, liver dysfunction, and physiological jaundice. Students were responsible for evaluating the biochemical data in each report, preparing retrospective case histories for hypothetical patients based on laboratory findings, and integrating laboratory data with potential clinical symptoms, physical examination findings, and provisional diagnoses. The standardized criteria for case design included prompts to describe the patient's age, sex, clinical symptoms, physical examination findings, and additional investigations. The facilitators monitored the sessions, answered questions, and provided comments. 

Assessment tool and scoring

A three-tiered assessment system was developed to evaluate the success of the educational method used. All three tests (pre-, post-, and delayed post-test) used an identical questionnaire to allow for direct comparison. The assessment was designed based on a blueprint to ensure content validity, covering all major topics: basic concepts of liver function, and interpretation of obstructive, hepatic, and hemolytic jaundice patterns. The questionnaire consisted of 10 multiple-choice questions (MCQs) testing foundational knowledge (1 mark each) and five short-answer questions (3 marks each) requiring interpretation of laboratory data and clinical correlation, for a total of 25 marks. Questions were mapped to four clinical categories - obstructive jaundice, hepatic jaundice, hemolytic jaundice, and mixed jaundice pattern - to allow for topic-wise analysis of student performance. The full questionnaire is provided in Appendix 1. The mapping of questions to clinical categories is provided in Appendix 2. MCQs were scored as correct (1 mark) or incorrect (0). Short-answer questions were scored using a detailed marking key, with points awarded for correct identification of the diagnosis, appropriate justification, and accurate interpretation of laboratory data. Scores were later converted to percentages for analysis.

To ensure objectivity in scoring, a detailed marking key was prepared for all short-answer questions. Two independent evaluators scored a random sample of 20 answer sheets. The inter-rater reliability was excellent (Intraclass Correlation Coefficient = 0.91), after which one evaluator scored the remaining scripts.

Feedback collection

In addition to quantitative assessments, qualitative feedback was obtained from the students and facilitators. A post-activity feedback questionnaire was administered to all 120 students immediately after they completed the post-test. The questionnaire was developed based on a literature review of similar educational interventions, and its content was reviewed by three medical education experts to establish content validity. It consisted of 10 items rated on a five-point Likert scale (1=Strongly Disagree to 5=Strongly Agree) covering themes of engagement, confidence, relevance, and perceived effectiveness of the learning method. The questionnaire also included two open-ended questions inviting students to provide qualitative comments on the strengths of the session and suggestions for improvement. The full feedback questionnaire is provided in Appendix 3. To assess the internal consistency of the Likert-scale items, a reliability analysis was performed, yielding a Cronbach's alpha of 0.82, indicating good reliability. Facilitators also provided informal verbal feedback, which was noted by the research coordinator.

Statistical analysis

Data were analyzed using SPSS version 27 (IBM Corp., Armonk, NY, USA). With 120 participants completing all assessments, the study had sufficient power to detect a moderate effect size (Cohen's d = 0.5) with 80% power at an alpha level of 0.05, based on a priori sample size calculation for paired t-tests. Descriptive statistics (mean, standard deviation) were calculated for test scores (expressed as percentages). To compare the mean scores across the three assessment time points (pre-test, post-test, and delayed post-test), paired samples t-tests were conducted for the two primary comparisons of interest: pre-test vs. post-test (to assess immediate knowledge gain) and pre-test vs. delayed post-test (to assess long-term knowledge retention). To account for these two comparisons, a Bonferroni correction was applied, with statistical significance set at p < 0.05. To estimate the magnitude of the intervention's effect, effect sizes (Cohen's d) were calculated for each pairwise comparison.

## Results

The results of this study demonstrated the usefulness of using retrospective case construction from hepatobiliary function test reports as a teaching method to improve laboratory data interpretation skills among first-year medical students. The findings are presented in three major domains: (1) analysis of pre-test, post-test, and delayed post-test scores; (2) evaluation of performance in specific areas of interpretation; and (3) qualitative feedback from students and facilitators. 

Comparative analysis of the test scores

A total of 120 students participated in pre-test, post-test, and delayed post-test assessments. Scores were analyzed to assess the improvements in interpretation skills and knowledge retention over time (Table 3). 

**Table 3:** Comparative Analysis of Assessment Scores (n=120) *Statistical significance was set at p < 0.05. SD: standard deviation;  df: degrees of freedom.

Assessment	Mean Score (%)	Comparison	Mean Difference (%)	t-value	df	p-value*	Effect Size (Cohen's d)
Pre-test	46.2	—	—	—	—	—	—
Post-test	78.4	Pre-test vs. Post-test	32.2	15.32	119	< 0.001	1.4 (Very Large)
Delayed Post-test	67.3	Pre-test vs. Delayed Post-test	21.1	8.47	119	< 0.001	0.8 (Large)

Pre-test Performance

The pre-test results revealed baseline unfamiliarity with hepatobiliary function tests. The average score was 46%, indicating a limited understanding of laboratory markers such as bilirubin, ALT, AST, and ALP. Many students struggled to correlate biochemical data with clinical conditions, highlighting a significant gap in their analytical skills. 

Post-test Performance

Post-test scores showed significant improvement, with an average score of 78%, indicating substantial gains in understanding and interpretation abilities immediately following the learning intervention. The improvement was statistically significant (p < 0.05), as determined using a paired t-test. The most notable increase was observed in questions related to obstructive jaundice, in which students demonstrated a clear understanding of the biochemical patterns and their clinical implications.

Delayed Post-test Performance

Four weeks after the intervention, the average score on the delayed post-test was 67%, indicating satisfactory knowledge retention. Although a slight decline was observed compared to the post-test score, the results still demonstrated a significant improvement over the pre-test scores. Long-term retention was highest for straightforward clinical scenarios, such as obstructive jaundice, whereas more complex cases, such as mixed hepatic jaundice patterns, showed a moderate decline. 

The mean difference between the pre- and post-test scores was statistically significant (p < 0.001), with a very large effect size (Cohen's d = 1.4), indicating the intervention had a substantial immediate impact. Similarly, the mean difference between the pre- and delayed post-test scores was also significant (p < 0.05, Cohen's d = 0.8), confirming a large effect and retention of knowledge over time.

Performance in specific areas of interpretation

To assess students' understanding of different clinical conditions, the post-test questionnaire was designed with questions mapped to four clinical categories: obstructive jaundice, hepatic jaundice, hemolytic jaundice, and mixed jaundice pattern (see Appendix 2 for question-topic mapping). For each category, accuracy was calculated as the percentage of students who correctly answered all questions pertaining to that specific topic. A summary of topic-wise performance is presented in Table 4.

**Table 4:** Topic-Wise Accuracy in Post-test Assessment (n=120)

Clinical Category	Number of Questions	Question Numbers	Average Accuracy (%)	Interpretation
Obstructive Jaundice	3	1, 7, 11	85	Exceptionally well performed
Hepatic Jaundice	3	4, 6, 12	75	Moderately well performed
Hemolytic Jaundice	3	2, 5, 13	70	Moderate difficulty
Mixed Jaundice Pattern	3	10, 14, 15	65	Most challenging

Students performed exceptionally well in the obstructive jaundice category, with an average post-test accuracy of 85% (Table 4). This suggests that the clarity of diagnostic markers, such as high direct bilirubin and ALP levels, facilitates learning. Performance in hepatic jaundice was moderate, with a post-test accuracy of 75% (Table 4). Students showed improvement but faced challenges in differentiating between hepatic and obstructive jaundice in borderline cases. For hemolytic jaundice, post-test accuracy was 70% (Table 4), reflecting the difficulty in correlating low direct bilirubin with high indirect bilirubin and understanding the absence of bilirubin in urine. The mixed jaundice pattern presented the most difficulty, with a post-test accuracy of 65% (Table 4). Students often confused overlapping biochemical markers, reflecting the complexity of these cases and the need for additional reinforcement. 

Qualitative feedback

Student Feedback

Student feedback was collected via the post-activity questionnaire. The quantitative Likert-scale data showed a high level of acceptance for the teaching method (Table 5). For example, over 85% of students agreed or strongly agreed that the activity increased their confidence in interpreting liver function tests. The open-ended responses were analyzed using a basic thematic analysis. Two researchers independently reviewed all comments to identify recurring themes and patterns. They then met to discuss their findings and reach a consensus on the main themes, which are presented in a synthesized form in Table 5. As noted in the table footnote, the representative comments are synthesized from common responses to illustrate recurring themes and are not mutually exclusive; a single student's comment could touch upon multiple themes. This analysis was descriptive and exploratory, intended to provide context for the quantitative findings. Common themes included appreciation for the clinical relevance of the exercise and the engaging, hands-on nature of the group work. No qualitative data analysis software was used for this descriptive analysis. The key findings are presented in Table 5.

**Table 5:** Summary of Qualitative Feedback From Students Percentages represent the proportion of students who selected 'Agree' or 'Strongly Agree' on the five-point Likert scale for questionnaire items corresponding to each theme. The representative comments are synthesized from common responses to the open-ended questions and illustrate recurring themes. As a single student's comment could touch upon multiple themes, the responses are not mutually exclusive.

Feedback Theme	Percentage of Students Agreeing	Representative Student Comment (Synthesized)
Increased Confidence	>85%	"I feel more confident in my ability to read and interpret liver function test reports."
Relevance to Clinical Practice	~90%	"This exercise helped me see the direct connection between biochemistry and patient diagnosis."
Engagement with Activity	>80%	"Constructing a case from the data was engaging and helped solidify the concepts."

Facilitator Feedback

Facilitators highlighted the strengths of the teaching method, including its ability to encourage active participation and critical thinking. However, they also noted challenges, such as varying levels of student preparation and the need for additional time to address complex cases. 

Statistical analysis of learning gain

The statistical analysis confirmed the positive impact of the teaching intervention. The mean difference between the pre- and post-test scores was statistically significant (p < 0.001). Similarly, the mean difference between the pre- and delayed post-test scores was also significant (p < 0.05), confirming retention of knowledge over time. However, a slight but significant decline was observed in the mean difference between post-test and delayed post-test scores, highlighting the need for reinforcement of concepts over a longer period of time. 

Correlation between retention and case complexity

Retention rates varied according to case complexity. In simple cases, retention was highest (85%) in cases with clear clinical markers such as obstructive jaundice. In complex cases, retention was lower (65%) in cases with overlapping markers, indicating the need for additional reinforcement and practice in complex clinical scenarios. 

## Discussion

The results of this study highlight the usefulness of retrospective case construction from hepatobiliary function test reports as an educational technique for improving laboratory data interpretation skills among first-year medical students. This section discusses the significance of the results in relation to the study objectives, evaluates the strengths and limitations of the strategy, and makes recommendations for further application and research. The significant improvement in post-test scores compared to pre-test scores demonstrated the usefulness of the teaching intervention in enhancing students' understanding of hepatobiliary function tests. An average post-test score of 78% demonstrated strong short-term knowledge acquisition, indicating that the organized approach combining didactic lectures, group-based teaching, and case construction was well received and effective. 

The highest performance was achieved in simple clinical situations, such as obstructive jaundice, when biochemical indicators, such as high direct bilirubin and alkaline phosphatase levels, showed a clear clinical pattern. This is consistent with recent research demonstrating that straightforward, well-defined clinical situations allow for better learning and application of knowledge in medical education [10]. Furthermore, the delayed post-test results, which averaged 67%, revealed satisfactory long-term retention of concepts. Although a slight decrease was observed compared to the post-test scores, delayed retention still demonstrated a significant improvement from the baseline pre-test levels. This suggests that the case-based approach not only stimulates rapid learning but also supports long-term retention. 

Retrospective case construction has proven to be a useful strategy for bridging the gap between theoretical biochemistry and its clinical applications. By challenging students to study biochemical data and develop hypothetical medical scenarios, this technique actively engages them in critical thinking and problem-solving. This is consistent with the concept of CBL, which promotes a student-centered and interactive learning environment [11]. The ability to connect laboratory findings with clinical symptoms is a key competency for medical professionals. The relevance of laboratory diagnostic tests to patient care has been emphasized [12], underscoring the need for practical experience in laboratory procedures during preclinical training. 

This study demonstrates how such exercises can be effectively introduced at the undergraduate level. This method's emphasis on small-group teaching further enhances its effectiveness. Facilitators provide individualized support, encouraging active participation and collaborative learning. Student feedback indicated strong levels of engagement and a sense of empowerment regarding their ability to evaluate and use biochemical data. The recall patterns observed in the delayed post-test underscore the importance of reinforcing complex concepts over time. While retention was strong for simple clinical scenarios such as obstructive jaundice, performance decreased in more complex cases such as those with mixed or borderline clinical patterns. This variability may be related to the inherent challenge of overlapping biochemical indicators, which typically require a deeper understanding of pathophysiological causes. 

The results of this study suggest that additional reinforcement, such as follow-up activities or periodic review sessions, may help reduce retention in difficult cases. Incorporating digital tools, simulations, or longitudinal case discussions may further enhance students' ability to retain and apply knowledge over time [13]. The findings highlight the advantages of interactive CBL over standard didactic teaching. The substantial gains in post-test scores and overwhelmingly positive student responses underscore the superiority of active learning methods in building conceptual knowledge and clinical reasoning. Compared to typical lecture-based approaches, retrospective case construction provides a more dynamic and engaging learning experience, allowing students to apply their theoretical knowledge to practical situations. Furthermore, the small-group structure used in this study provided a supportive learning environment in which students felt comfortable asking questions and exploring complex ideas [14]. The individualized attention from the facilitators likely contributed to the high levels of confidence that students reported in their ability to evaluate laboratory data [15]. 

The study's multi-phase design, including pre-, post-, and delayed post-test assessments, allowed for a thorough examination of learning outcomes and retention. The use of small-group teaching allowed for discussions and customized feedback, while the inclusion of a varied range of hepatobiliary cases meant that students were exposed to various clinical problems. Structured feedback from both students and facilitators complemented the findings, revealing insights into the strategy's success and areas for improvement. Positive student comments on interest, confidence, and relevance to clinical practice highlight the importance of implementing comparable strategies across a comprehensive medical curriculum. 

Despite its advantages, this study had several limitations that should be considered when interpreting the findings. First, the relatively small sample size of 120 students from a single institution may limit the generalizability of our findings to other settings or student populations. Second, the absence of a control or comparison group means we cannot definitively attribute all observed improvements solely to the intervention; factors like maturation or concurrent learning cannot be ruled out. Third, the use of identical pre-, post-, and delayed post-test questionnaires introduces the potential for a "testing effect," where students may have improved simply due to repeated exposure to the same test items. Fourth, we did not adjust for students' baseline academic performance, which could have influenced their ability to benefit from the intervention. Fifth, while we used robust statistical tests, the reliance on p-values alone can be misleading; we have therefore included effect sizes to provide a clearer measure of the educational impact. Finally, the potential heterogeneity of facilitator capability, although minimized through training, may have influenced the small-group dynamics and learning outcomes in different groups. Furthermore, delayed post-test scores may have been altered by external influences, such as additional exposure to biochemical principles or clinical settings during the four-week interval.

The results of this study have substantial implications for medical education, particularly in the early years of training. The retrospective case design provides a practical and entertaining approach to teaching biochemistry, helping students bridge the gap between core science and clinical practice. This strategy prepares students for advanced clinical training by developing their critical thinking and clinical reasoning skills. The results also highlight the value of early exposure to clinical situations, which can help students gain a greater understanding of the relevance of basic science to patient care. Incorporating such tactics into the medical curriculum is crucial. This could enhance students' preparation for clinical rotations and future medical practice. Future studies should examine the scalability of this strategy across a wider and more diverse student population. Comparative research comparing the effectiveness of retrospective case design with other active learning methods, such as problem-based or simulation-based learning, could provide additional insight into its relative merits. Longitudinal studies evaluating the impact of this strategy on students' clinical performance in subsequent years of training would also be helpful. Furthermore, integrating digital tools and simulations into the learning process could increase the accessibility and scalability of this method, especially in resource-constrained situations.

## Conclusions

This quasi-experimental study demonstrates that retrospective case construction from hepatobiliary function test reports is an effective pedagogical tool for improving laboratory data interpretation skills among first-year medical students. The significant improvement from pre-test to post-test scores indicates substantial immediate knowledge gain following the educational intervention. Furthermore, the sustained improvement observed at four weeks confirms satisfactory knowledge retention of the concepts taught.

Student feedback reinforced these quantitative findings, with the majority of participants reporting increased confidence in interpreting liver function tests and acknowledging the clinical relevance of the exercise. The highest retention rates were observed for straightforward clinical patterns such as obstructive jaundice, while more complex cases with overlapping biochemical markers showed comparatively lower retention, indicating the need for additional reinforcement of challenging concepts.

These findings suggest that retrospective case construction can serve as a valuable recurrent activity in the early medical curriculum to bridge the gap between foundational biochemistry and clinical practice.

## Appendices

Appendix 1: Assessment questionnaire (pre-test, post-test, and delayed post-test)

Instructions: This questionnaire assesses your understanding of hepatobiliary function tests. Please answer all questions to the best of your ability. The test is divided into two parts: Multiple Choice Questions (MCQs) and Short Answer Questions (SAQs).

Total Marks: 25 (10 MCQs x 1 mark each = 10 marks; 5 SAQs x 3 marks each = 15 marks)

Part A: Multiple Choice Questions (1 Mark Each)

Choose the single best answer for each question.

1. A patient presents with dark urine, pale stools, and pruritus. Laboratory investigations reveal a markedly elevated serum alkaline phosphatase (ALP) level, with mildly elevated ALT and AST. Direct bilirubin is significantly higher than indirect bilirubin. These findings are most consistent with: a) Hemolytic jaundice b) Hepatic (hepatocellular) jaundice c) Obstructive (cholestatic) jaundice d) Physiological jaundice of the newborn

2. Which of the following liver function test patterns is characteristic of hemolytic jaundice? a) Increased direct bilirubin, increased ALP, normal ALT b) Increased indirect bilirubin, normal ALP, normal ALT, increased urinary urobilinogen c) Increased direct and indirect bilirubin, grossly elevated ALT, normal ALP d) Increased direct bilirubin, increased ALP, grossly elevated ALT

3. The primary enzyme marker indicative of biliary obstruction and cholestasis is: a) Alanine aminotransferase (ALT) b) Aspartate aminotransferase (AST) c) Alkaline phosphatase (ALP) d) Gamma-glutamyl transferase (GGT)

4. In a patient with viral hepatitis causing significant hepatocellular injury, which of the following patterns is most likely to be seen? a) A disproportionate rise in ALP compared to ALT/AST b) A predominant rise in indirect bilirubin c) A marked elevation in ALT and AST (often >10x upper limit of normal) d) Normal liver enzyme levels with isolated hyperbilirubinemia

5. The presence of bilirubin in urine (bilirubinuria) indicates an elevation of which fraction of bilirubin in the blood? a) Indirect (unconjugated) bilirubin b) Direct (conjugated) bilirubin c) Both direct and indirect bilirubin equally d) Delta bilirubin

6. A 45-year-old male with a history of alcohol abuse presents with jaundice and ascites. Which set of lab findings is most suggestive of alcoholic liver disease? a) ALT > AST, with a ratio of 2:1 b) AST > ALT, with a ratio of 2:1 c) Markedly elevated ALP with normal transaminases d) Isolated elevation of indirect bilirubin

7. Which of the following findings is more characteristic of obstructive jaundice than of hepatic jaundice? a) Markedly elevated ALT b) Prolonged prothrombin time that corrects with vitamin K c) Reduced serum albumin d) Markedly elevated AST

8. In a neonate with physiological jaundice, the hyperbilirubinemia is predominantly: a) Conjugated (direct) b) Unconjugated (indirect) c) Delta bilirubin d) Mixed conjugated and unconjugated

9. Which enzyme is also elevated in obstructive jaundice but is more specific for liver pathology when used to confirm the hepatic origin of an elevated ALP? a) ALT b) AST c) Gamma-glutamyl transferase (GGT) d) Lactate dehydrogenase (LDH)

10. A patient with jaundice has a total bilirubin of 5.0 mg/dL, with a direct fraction of 1.0 mg/dL. Urinalysis shows no bilirubin but increased urobilinogen. This pattern is consistent with: a) Obstructive jaundice b) Hepatocellular jaundice c) Hemolytic jaundice d) Choledocholithiasis

Part B: Short Answer Questions (3 Marks Each)

Answer the following questions in the space provided. For questions involving test reports, interpret the data and provide a provisional diagnosis with brief justification.

11. A 55-year-old male presents with jaundice, right upper quadrant pain, and fever. His laboratory report shows the following: Total Bilirubin: 6.2 mg/dL Direct Bilirubin: 4.8 mg/dL Indirect Bilirubin: 1.4 mg/dL ALT: 85 U/L (Normal < 40) AST: 70 U/L (Normal < 40) ALP: 450 U/L (Normal 30-120)

What is the most likely provisional diagnosis based on this report? Justify your answer by interpreting the pattern of bilirubin and enzyme levels.

12. A 25-year-old female with a history of fatigue and malaise for two weeks undergoes investigations. Her report is as follows: Total Bilirubin: 3.5 mg/dL Direct Bilirubin: 1.8 mg/dL Indirect Bilirubin: 1.7 mg/dL ALT: 950 U/L (Normal < 40) AST: 1100 U/L (Normal < 40) ALP: 110 U/L (Normal 30-120)

What is the most likely provisional diagnosis? Justify your answer.

13. A blood report shows the following findings in a young adult: Total Bilirubin: 2.8 mg/dL Direct Bilirubin: 0.6 mg/dL Indirect Bilirubin: 2.2 mg/dL ALT: 30 U/L (Normal < 40) AST: 35 U/L (Normal < 40) ALP: 85 U/L (Normal 30-120) Peripheral blood smear shows spherocytes. Urine examination shows no bilirubin, but increased urobilinogen.

What is the most likely diagnosis? Explain the pathophysiological basis for the elevated indirect bilirubin and the absence of bilirubin in the urine.

14. Briefly explain the clinical significance of fractionating bilirubin into direct and indirect components. What does an elevated direct bilirubin indicate versus an elevated indirect bilirubin?

15. A 60-year-old chronic alcoholic presents with jaundice, ascites, and pedal edema. His lab report is as follows: Total Bilirubin: 4.1 mg/dL Direct Bilirubin: 2.5 mg/dL Indirect Bilirubin: 1.6 mg/dL ALT: 65 U/L AST: 120 U/L (Note: AST > ALT) ALP: 150 U/L Serum Albumin: 2.5 g/dL (Normal 3.5-5.0) Prothrombin Time: Prolonged

Based on the entire clinical picture and lab data, what is the most likely diagnosis? What do the low albumin and prolonged PT suggest?

Appendix 2: Mapping of assessment questions to clinical categories

Mapping of assessment questions to various clinical categories is listed in Table 6.

**Table 6:** Mapping of Assessment Questions to Clinical Categories MCQ: multiple choice question; SAQ: short answer question; ALP: alkaline phosphatase; ALT: alanine aminotransferase; AST: aspartate aminotransferase; GGT: gamma-glutamyl transferase; PT: prothrombin time.

Question Number	Question Type	Clinical Category	Topic Assessed
Q1	MCQ	Obstructive Jaundice	Identification of obstructive pattern (dark urine, pale stools, elevated ALP, high direct bilirubin)
Q2	MCQ	Hemolytic Jaundice	Characteristic pattern of hemolytic jaundice (indirect hyperbilirubinemia, normal enzymes, increased urobilinogen)
Q3	MCQ	General	Primary enzyme marker for biliary obstruction (ALP)
Q4	MCQ	Hepatic Jaundice	Pattern in viral hepatitis/hepatocellular injury (markedly elevated transaminases)
Q5	MCQ	Hemolytic Jaundice	Relationship between bilirubinuria and bilirubin fractions
Q6	MCQ	Hepatic Jaundice	Alcoholic liver disease (AST > ALT ratio)
Q7	MCQ	Obstructive Jaundice	Differentiating feature of obstructive vs. hepatic jaundice (PT correction with vitamin K)
Q8	MCQ	General	Physiological jaundice of newborn (unconjugated hyperbilirubinemia)
Q9	MCQ	General	GGT as confirmatory enzyme for liver origin of elevated ALP
Q10	MCQ	Mixed Pattern	Interpretation of mixed biochemical findings (hemolytic pattern with normal enzymes)
Q11	SAQ	Obstructive Jaundice	Case-based interpretation: obstructive pattern with elevated ALP, direct bilirubin, and mild transaminitis
Q12	SAQ	Hepatic Jaundice	Case-based interpretation: hepatocellular pattern with markedly elevated transaminases
Q13	SAQ	Hemolytic Jaundice	Case-based interpretation: indirect hyperbilirubinemia, spherocytes, absent urine bilirubin, increased urobilinogen
Q14	SAQ	General	Clinical significance of bilirubin fractionation (direct vs. indirect)
Q15	SAQ	Mixed Pattern	Case-based interpretation: chronic liver disease with mixed features (AST>ALT, low albumin, prolonged PT)

Appendix 3: Post-activity feedback questionnaire

Instructions: Please indicate your level of agreement with the following statements about today's learning activity ("Retrospective Case Construction from Hepatobiliary Function Test Reports"). Mark only one option per statement. Your responses are anonymous and will be used to improve future educational sessions (Table 7).

**Table 7:** Post-activity Feedback Questionnaire Items The questionnaire was administered immediately after the post-test on Day 1. Responses were anonymous. Likert-scale items were scored from 1 (Strongly Disagree) to 5 (Strongly Agree). ALP: alkaline phosphatase

S. No.	Statement	1	2	3	4	5
1	The objectives of the session were clear to me.	☐	☐	☐	☐	☐
2	The small-group activity was engaging and helped me learn.	☐	☐	☐	☐	☐
3	Constructing a case from the lab reports was a useful way to understand the concepts.	☐	☐	☐	☐	☐
4	I feel more confident in my ability to read and interpret liver function test reports after this session.	☐	☐	☐	☐	☐
5	This exercise helped me see the direct connection between biochemistry and patient diagnosis.	☐	☐	☐	☐	☐
6	The facilitator provided helpful guidance during the group activity.	☐	☐	☐	☐	☐
7	This session should be continued for future batches of first-year students.	☐	☐	☐	☐	☐
8	I was able to correlate the biochemical markers (e.g., bilirubin, ALP) with clinical conditions.	☐	☐	☐	☐	☐
9	The session was well-organized and the time allocated was sufficient.	☐	☐	☐	☐	☐
10	This type of learning is more effective than a traditional lecture alone for this topic.	☐	☐	☐	☐	☐

Open-Ended Questions:

What were the main strengths of this learning session?

Do you have any suggestions for how this session could be improved in the future?

Thank you for your participation!
